# *Aconitum lycoctonum* L. (Ranunculaceae) mediated biogenic synthesis of silver nanoparticles as potential antioxidant, anti-inflammatory, antimicrobial and antidiabetic agents

**DOI:** 10.1186/s13065-023-01047-5

**Published:** 2023-09-28

**Authors:** Zia ur Rehman Khan, Nasir Assad, Muhammad Naeem-ul-Hassan, Muhammad Sher, Fatema Suliman Alatawi, Mohsen Suliman Alatawi, Awatif M. E. Omran, Rasha M. A. Jame, Muhammad Adnan, Muhammad Nauman Khan, Baber Ali, Sana Wahab, Sarah Abdul Razak, Muhammad Ammar Javed, Alevcan Kaplan, Mehdi Rahimi

**Affiliations:** 1 Institute of Chemistry, University of Sargodha, Sargodha, 40100 Pakistan; 2 Department of Biochemistry, Faculty of Science, University of Tabuk, Tabuk, Saudi Arabia; 3 Department of Pediatrics, College of Medicine, King Saud Bin Abdulaziz University for Health Sciences, Riyadh, Saudi Arabia; 4 Department of Chemistry, Faculty of Science, University of Tabuk, Tabuk, Saudi Arabia; 5 Department of Chemistry, Faculty of Education, University of Dalanj, Dalanj, Sudan; 6 Department of Chemistry, Islamia College Peshawar, Peshawar, 25120 Pakistan; 7 Department of Botany, Islamia College Peshawar, Peshawar, 25120 Pakistan; 8 Department of Plant Sciences, Quaid-I-Azam University, Islamabad, Pakistan; 9 Institute of Biological Sciences, Faculty of Science, Universiti Malaya, 50603 Kuala Lumpur, Malaysia; 10 Institute of Industrial Biotechnology, Government College University, Lahore, 54000 Pakistan; 11 Department of Crop and Animal Production, Sason Vocational School, Batman University, 72060 Batman, Turkey; 12 Department of Biotechnology, Institute of Science and High Technology and Environmental Sciences, Graduate University of Advanced Technology, Kerman, Iran

**Keywords:** *A. lycoctonum*, Antibacterial, Antidiabetic, Anti-inflammatory, Antioxidant, Silver nanoparticles

## Abstract

In this study, a polar extract of *Aconitum lycoctonum* L. was used for the synthesis of silver nanoparticles (AgNPs), followed by their characterization using different techniques and evaluation of their potential as antioxidants, amylase inhibitors, anti-inflammatory and antibacterial agents. The formation of AgNPs was detected by a color change, from transparent to dark brown, within 15 min and a surface resonance peak at 460 nm in the UV–visible spectrum. The FTIR spectra confirmed the involvement of various biomolecules in the synthesis of AgNP*s*. The average diameter of these spherical AgNP*s* was 67 nm, as shown by the scanning electron micrograph. The inhibition zones showed that the synthesized nanoparticles inhibited the growth of Gram-positive and negative bacteria. FRAP and DPPH assays were used to demonstrate the antioxidant potential of AgNP*s*. The highest value of FRAP (50.47% AAE/mL) was detected at a concentration of 90 ppm and a DPPH scavenging activity of 69.63% GAE was detected at a concentration of 20 µg/mL of the synthesized AgNP*s*. 500 µg/mL of the synthesized AgNP*s* were quite efficient in causing 91.78% denaturation of ovalbumin. The AgNPs mediated by *A. lycoctonum* also showed an inhibitory effect on α-amylase. Therefore, AgNPs synthesized from *A. lycoctonum* may serve as potential candidates for antibacterial, antioxidant, anti-inflammatory, and antidiabetic agents.

## Introduction

Nanotechnology is an emerging field of research that is attracting a lot of attention worldwide. This technology involves the production of nanomaterials and nanoparticles (NP*s*) that can be used in many different fields, such as electrochemistry, catalysis, sensing, pharmaceuticals, biomedicine, cosmetics, food technology, etc. [1]. Depending on their size and shape, NPs have better physical properties than bulk molecules [2]. NP*s* composed of metals and metal oxides are the subject of numerous studies in the fields of science and technology due to their potential applications [3]. They are characterized by high surface-to-volume ratio and a low tendency to aggregate in aqueous solutions [4]. As a result, metal and metal oxide nanoparticles exhibit high antibacterial activity [5–8].

Conventional methods often require the use of potentially hazardous and costly chemicals [9]. The preparation of metal and metal oxide nanoparticles by biogenic route using aqueous plant extracts and microorganisms has gained popularity in recent years because it offers many advantages over conventional methods, such as low environmental impact, high stability, adaptability to therapeutic purposes, biocompatibility, and low cost [10, 11]. Many different types of metal and metal oxide NP have been synthesized so far, often using plant extracts, microorganisms, etc. [12].

Special attention was paid to the evaluation of antioxidant and reducing phytochemicals from plants or a microorganism-mediated bioreduction process as a cost effective and environmentally friendly method to produce AgNP*s*. In biosynthesis, the shape and size of AgNP*s* are mainly determined by the materials used for their production [13]. The biosynthesis of AgNP*s* by plants is fast, simple, and highly efficient. Plants contain a variety of metabolites, including those that can degrade and stabilize NP*s*. These metabolites include phenols, carboxylic acids, ketones, amides, aldehydes, and proteins. Almost all components of plants, including leaves, seeds, roots, and flowers, have been screened for active chemicals for the production of AgNP*s* [14]. Recent research on the biosynthesis of AgNP*s* has shown that the focus has shifted to the use of medicinal plants for the biosynthesis of nanoparticles. Various parts of medicinal plants have the best potential to attenuate and stabilize AgNP*s* due to their high concentration of reducing components (H^+^) [15]. Botanical chemicals with proven antimicrobial, anticancer, and neuroprotective activities are obtained from medicinal plants. Therefore, the inclusion of medicinal plants in biosynthesis development can improve the biological properties of NP*s*, which is more than can be said for a green chemical method alone [16].

Here we report the biogenic synthesis of AgNP*s* from a polar extract of *A. lycoctonum*, a member of the genus *Aconitum* found in Chitral (Pakistan), Kashmir, and India. *A. lycoctonum* is used by the locals in various medicinal applications due to its analgesic, anti-inflammatory, and immunomodulatory properties. The synthesized AgNP*s* were thoroughly characterized by UV–visible spectroscopy, FTIR spectroscopy, SEM, and EDX. The antioxidant properties of the AgNP*s* synthesized from *A. lycoctonum* were evaluated by 2, 2-diphenyl-1-picrylhydrazyl (DPPH) assay and FRAP assay. In addition, the antimicrobial properties against both Gram-positive and Gram-negative bacteria were evaluated using the well diffusion method. The synthesized AgNP*s* were also evaluated for their potential anti-inflammatory and anti-diabetic activities. Overall, this study demonstrates the adaptability and efficacy of AgNP*s* produced from *A. lycoctonum* and paves the way for their future use as therapeutic agents to combat major health problems.

## Materials and methods

### Materials

The plant sample was collected from Muzaffarabad and Poonch division of Azad Jammu & Kashmir, Pakistan. Plant identification and authentication was done by a taxonomist from at the Department of Botany, Sargodha University, Sargodha, Pakistan. Silver nitrate (AgNO_3_) (Merck, Germany), DPPH (Sigma-Aldrich®), α-amylase (UNI-_CHEM_®), ethanol (Sigma- Aldrich®), diclofenac sodium (Acros Organics) Thermo Fisher Scientific US and *n*-hexane (EMSURE ACS, Malaysia) were purchased from local market, and was of analytical grade. Nutrient agar (Oxoid) and bacterial strains *Escherichia coli* (ATCC 10536), *Listeria monocytogenes* (ATCC 13932), *Staphylococcus epidermidis* (ATCC 12228), and *Pseudomonas aeruginosa* (ATCC 10145) were available from the Biochemistry Laboratory of the Institute Chemistry, Sargodha University, Punjab Pakistan. Deionized water was used for preparation of solutions and extractions.

### Extraction of polar extracts

The polar extract from the root of *A. lycoctonum* was extracted by the conventional method. The tuberous roots were washed with purified water (DW) to remove dirt and other impurities, and then dried in the shade at 25 ℃ for 10 days. After drying, the roots were ground through a grinder and then passed through a fine sieve. To prepare the root powder solution, 5 g of fine root powder was thoroughly mixed with 100 mL of DW in a 250 mL volumetric flask. This solution was then stirred on a stir plate at room temperature for 5 h. The solution was filtered with Whatman filter paper, and the filtrate was washed in *n*-hexane. After washing with *n*-hexane, two layers were formed i.e., a polar and a nonpolar layer, which were separated with a separating funnel. The nonpolar layer was discarded, while the polar layer was poured into a Petri dish and dried for 24 h at 45 °C in an air drying oven. After drying, the polar extract was separated from the Petri dish and sealed in an Eppendorf tube for further use.

### Synthesis of *A. lycoctonum *mediated AgNP*s*

The solution of *A. lycoctonum (*1 mM) was freshly prepared by dissolving 17.0 mg of polar extract in 100 mL of DW and a solution of AgNO_3_ (1 mM) was also prepared by dissolving 17.0 mg of AgNO_3_ in 100 mL of DW. The solutions were mixed and the reaction mixture was irradiated with sunlight and the color changes were observed over a period of 15 min.

### Characterization

The color of AgNPs changes from yellowish brown to reddish brown and finally to reddish black. These changes were studied using a UV–vis spectrophotometer (UV-1700 Pharmspec, Shimadzu) in the range of 800–200 nm by detecting the absorption peaks over a period of 15 min (0, 1, 5, 10, and 15 min).

The compatability of AgNPs mediated by *A. lycoctonum* was investigated by FTIR spectroscopy. This also detects the vibrations of stretching and bending bonds. Infrared spectra were obtained with a spectrometer (Schimadzu FTIR 8400S) using the KBr-pellet technique with a sampling scanning range of 4000–500 cm^−1^. The size and shape of the synthesized AgNP*s* were examined using a scanning electron microscope (SEM). Samples (microtomes), were analyzed using carbon sample tubes (carbon sticker No. G3347) from Plano (Wetzlar, Germany) at SEM. Energy dispersive X-ray (EDX) analysis was used to analyze, the X-rays produced by the sample when bombarded with an electron beam to characterize the elemental structure of the volume. The Malvern Zetasizer Nano ZS was used to calculate the hydrodynamic diameter of the nanoparticles. A He–Ne laser with a wavelength of 633 nm was used for the measurements. The sample suspension was filtered through a Whatman filter (0.2 µm) to remove all solid impurities. The filtrate was stored in a solvent-resistant microcuvette with a 10 mm path length. Prior to analysis, the sample was heated to 25 °C in the analyzer for 2 min in the analyzer. Mean standard deviations were given for size studies. Malvern’s Zetasizer software was used to analyze the data.

### Antibacterial activity

The following protocol from [17] was adopted with some modifications for the assay of antibacterial activity of AgNP*s*: 6.3 g of nutrient agar (Oxoid) was dissolved in 120 mL of DW. The agar solution and Petri dishes were then placed in an autoclave at 121 °C for 15 min. After sterilization, the medium was cooled to 50 °C, then 25 mL of agar solution was poured into each Petri dish and allowed to solidify for 20 min. Pure cultures of the organism were subcultured on nutrient agar at 35 °C in a rotary shaker at 200 rpm. Then, the desired bacterial strains, *Escherichia coli* (ATCC 10536), *Listeria monocytogenes* (ATCC 13932), *Staphylococcus epidermidis* (ATCC 12228), and *Pseudomonas aeruginosa* (ATCC 10145) were introduced into each Petri dish using stick plate technique. After spreading, five wells were formed with a cork borer and labeled alphabetically. Then, 30 µL of DW (negative control), 30 µL of ceftriaxone sodium (1 mg/mL) (positive control), 30 µL (1 mg/mL) of AgNPs, 30 µL (1 mg/mL) of plant extract, and 30 µL (1 mg/mL) of AgNO_3_ solution were added to each well, and the Petri dishes were then placed in an incubator at 37 °C for 24 h. The whole process was performed in laminar flow cabinet and sterile laboratory conditions. Antibacterial activity was repeated in triplicate and values were expressed as mean ± standard deviation.

### Antioxidant activity

#### Ferric reducing antioxidant power (FRAP) assay

Using a FRAP assay, that the root extract of *A. lycoctonum* which induces AgNP*s*, was found to have significant antioxidant activity. The FRAP assay was performed according to the protocol of Benzie and Strain (1996) with slight modifications [18]. DW was used to dilute AgNP*s* to levels between 10 µg and 50 µg/L. The ascorbic acid solution was used as a standard in FRAP assays. Reference standard solutions were prepared at concentrations of 10 µg/L, 20 µg/L, 30 µg/L, 40 µg/L, and 50 µg/L. To prepare a phosphate buffer (pH 6.6), 0.2 g of potassium chloride, 1.44 g of disodium hydrogen phosphate, 8 g of sodium chloride, and 0.24 g of potassium dihydrogen phosphate were mixed in 500 mL of DW respectively, and then added HCl was added until the pH 6.6 was reached. To the AgNP*s* samples and controls, 2.5 mL of phosphate buffer (pH 6.6) and 2.5 mL of 1% K_3_Fe(CN)_6_ were added. After vortexing for 5 min, the mixture was incubated at 50 °C for 20 min. After incubation, the mixture was treated with 10% TCA in 2.5 mL and centrifuged (3000 rpm for 10 min). 2.5 mL of DW was added to the collected supernatant from the centrifuge. A colored solution was obtained by adding 0.51 mL of 0.1% ferric chloride to the mixture. A UV–Visible spectrophotometer set to 711 nm was used to analyze the samples and reference standard solutions. The FRAP assay was performed in triplicate and the values were expressed as mean ± standard deviation.

#### 2,2-Diphenyl-1-picrylhydrazyl (DPPH) radical scavenging capacity assay

The scavenging activity of AgNP*s* was evaluated using the DPPH radical as a free radical model, as previously described by [19]. An ethanolic solution (99.8% ethanol) of DPPH (4 mg/100 mL) was prepared and stored in a cool place. A series of plant extract mediated AgNP*s* (1, 2, 3, 4, 5 mL) were prepared and made up to 10 mL with ethanol. Then 1 mL of each sample was mixed with 3 mL of DPPH and ethanol was added to make up the reaction mixture to 10 mL. Gallic acid was used as a reference standard. The absorbance of the solutions was measured at 517 nm after incubation at room temperature in the dark for 30 min. The percentage of inhibition was estimated by substituting these values into the following formula: The DPPH assay was repeated in triplicate and the values were expressed as mean ± standard deviation.

$$\mathrm{\% inhibition}= (\mathrm{Abs Crl }-\mathrm{ Abs Spl}) /\mathrm{ Abs Crl }\times 100$$

Here, Abs Crl is the absorbance of control and Abs Spl is the absorbance of AgNP*s* and DPPH.

### Anti-inflammatory activity

#### Inhibition of the protein denaturation

The anti-inflammatory effects of AgNPs mediated by the extract from the root of *A. lycoctonum* were determined by denaturing the proteins according to the protocol previously described by [20] with slight modifications. The solutions of *A. lycoctonum* L. root extract-mediated AgNPs (0.1 mg/mL to 0.5 mg/mL) were mixed with 2.8 mL of phosphate-buffered saline (PBS) (pH 6.4) and 0.2 mL of fresh egg albumin solution to obtain a final volume of 5 mL. After 20 min of incubation at 37 °C, mixtures were heated for 5 min at 70 °C. Diclofenac sodium (0.1 mg/mL to 0.5 mg/mL) was used as a reference drug and treated similarly for absorbance determination. A control solution was prepared by mixing 2.8 mL of PBS (pH 6.4), and 0.2 mL of egg albumin solution and brought to total volume of 5 mL by addition of DW and treated similarly to the sample solution. A UV–Vis spectrophotometer was used to measure turbidity at a wavelength of 660 nm. A phosphate buffer was used as a control. The following formula was used to determine the inhibition of protein denaturation:

$$\mathrm{Inhibition\, of\, protein \,denaturation }\left(\mathrm{\%}\right)= 100 \times [1-\mathrm{ Absorbance }(\mathrm{sample})/\mathrm{Absorbance }(\mathrm{Control})]$$

### Anti-diabetic activity

#### α-Amylase inhibition by starch hydrolysis

Starch hydrolysis was measured by measuring the zone of inhibition on Petri dishes the protocol described in [21] with few modifications. A cork borer was used to make five wells on agar plates containing 1% (w/v) starch distributed in 1.5% agar. An α-amylase solution (EC 3.2.1.1) of *Aspergillus oryzae* equivalent to 2 U/mL in phosphate buffer (pH 6.9) was prepared and added to the well (A) as a control. The other wells with the different dilutions were mixed with the enzyme (B) standard (Acarbose), (C) AgNP*s*, (D) plant extract, (E) negative control. The plates were left at 25 °C for 3 days then stained with iodine and left for 15 min. After 72 h of incubation at 37 °C, the starch was stained with iodine solution (0.5 mM I_2_ in 3% KI) to measure α-amylase activity. The inhibitory effect was measured by measuring the radius of the hydrolyzed zone around the wells. The results were expressed as a percentage:

$$\mathrm{\% \alpha }-\mathrm{amylase \,inhibition}=\{(\mathrm{diameter \,of \,control }-\mathrm{ diameter \,of \,sample})/\mathrm{diameter \,of \,the \,control }\}\times 100$$

#### Digestive enzymes inhibition and kinetics

With some modifications to the regular working protocol previously described by [22] with some modifications, the amylase inhibition experiment was performed. 250 µL AgNP*s* solutions (10 mg/mL to 30 mg/mL) were mixed with 250 µL amylase (0.4 U/mL, in 0.02 M phosphate buffer solution (PBS) pH 6.9, containing 0.06 M sodium acetate). After a 10 min incubation at 37 °C, 250 µL of soluble starch 1% (w/v) (in 0.02 M PBS, pH 6.9) was added, followed by another 30 min of incubation. The reaction was stopped by heating the mixture in a boiling water bath for 10 min after adding 250 µL of dinitrosalicylic acid color reagent (DNS 96 mM, 30% Na–K tartrate, 0.4 M NaOH). After cooling to room temperature and dilution with 2 mL PBS, absorbance was measured at 540 nm. The percentage (%) of inhibition was determined using the following formula:

$$\mathrm{Percentage\, of \,inhibition }= (\mathrm{K}-\mathrm{S}) /\mathrm{ K }\times 100$$

K = Absorption of negative controls; S = Absorbance of sample/Absorbance of positive control.

## Results and discussion

### Synthesis of *A. lycoctonum* mediated AgNPs

In this framework, hydrogenated electrons are generated by exposing the AgNO_3_ solution to diffused sunlight. These electrons can be used to reduce the monovalent silver cations (Ag^+^) to the zerovalent silver (Ag^0^) [23]. The Ag^0^ atoms produced in this way are usually of nanometer size.

**Fig. 1:**
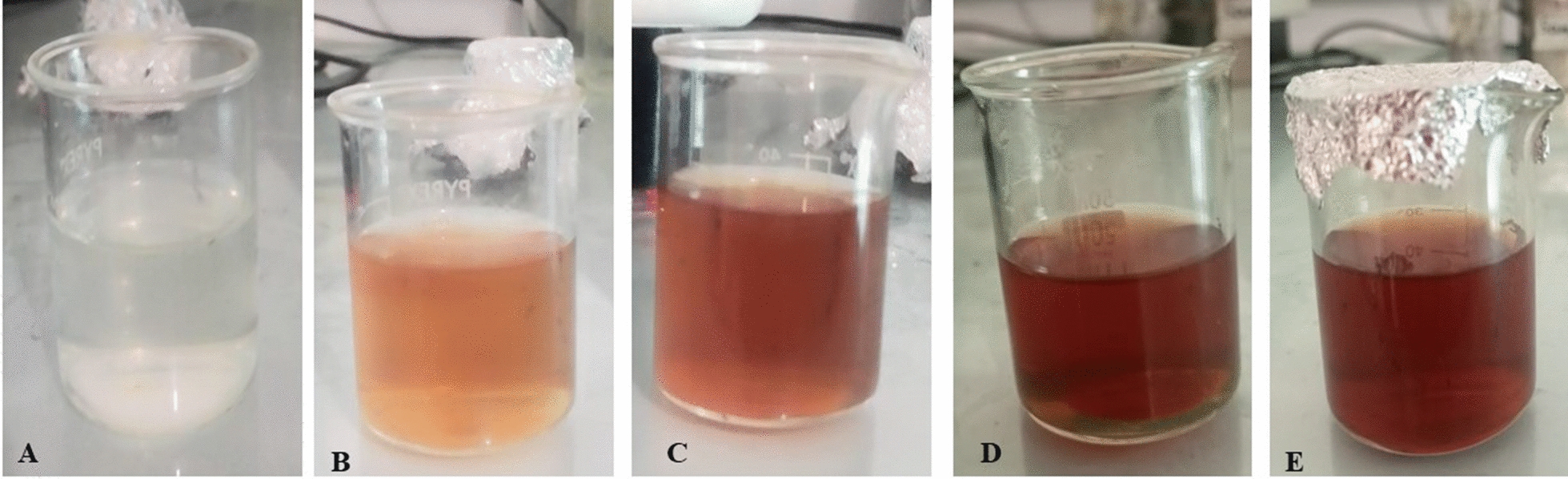
A depiction of the color by exposing the *A. lycoctonum*-AgNO_3_ mixture is exposed to diffused sunlight; **A** 0 min, **B** Exposed to sunlight for 1 min, **C** 5 min, **D** 10 min, and **E** 15 min

The synthesis of AgNP*s* mediated by *A. lycoctonum* was carried out using a 1 mM solution of AgNO_3_. After the reactants were combined, the polar extract of *A. lycoctonum* interacted with the Ag^+^ in the AgNO_3_ solution to form a complex of Ag and *A. lycoctonum*. The polar extract of *A. lycoctonum* converted the Ag^+^ in the complex to an Ag- *A. lycoctonum* precursor when the mixture was exposed to diffused sunlight. By observing the color shift of the reaction mixture over time, we were able to follow the evolution of AgNP*s* during irradiation of the (Ag)-*A. lycoctonum* complex. In less than 60 s, the color of mixture of the *A. lycoctonum* and AgNO_3_ changed from clear to reddish brown and then to dark brown, indicating that all Ag^+^ had been reduced [24]. Figure 1 illustrates the monitoring of the synthesis of AgNPs mediated by *A. lycoctonum* by the change in color over time, with Ag content causing a color change.

### UV–Visible spectroscopy

**Fig. 2:**
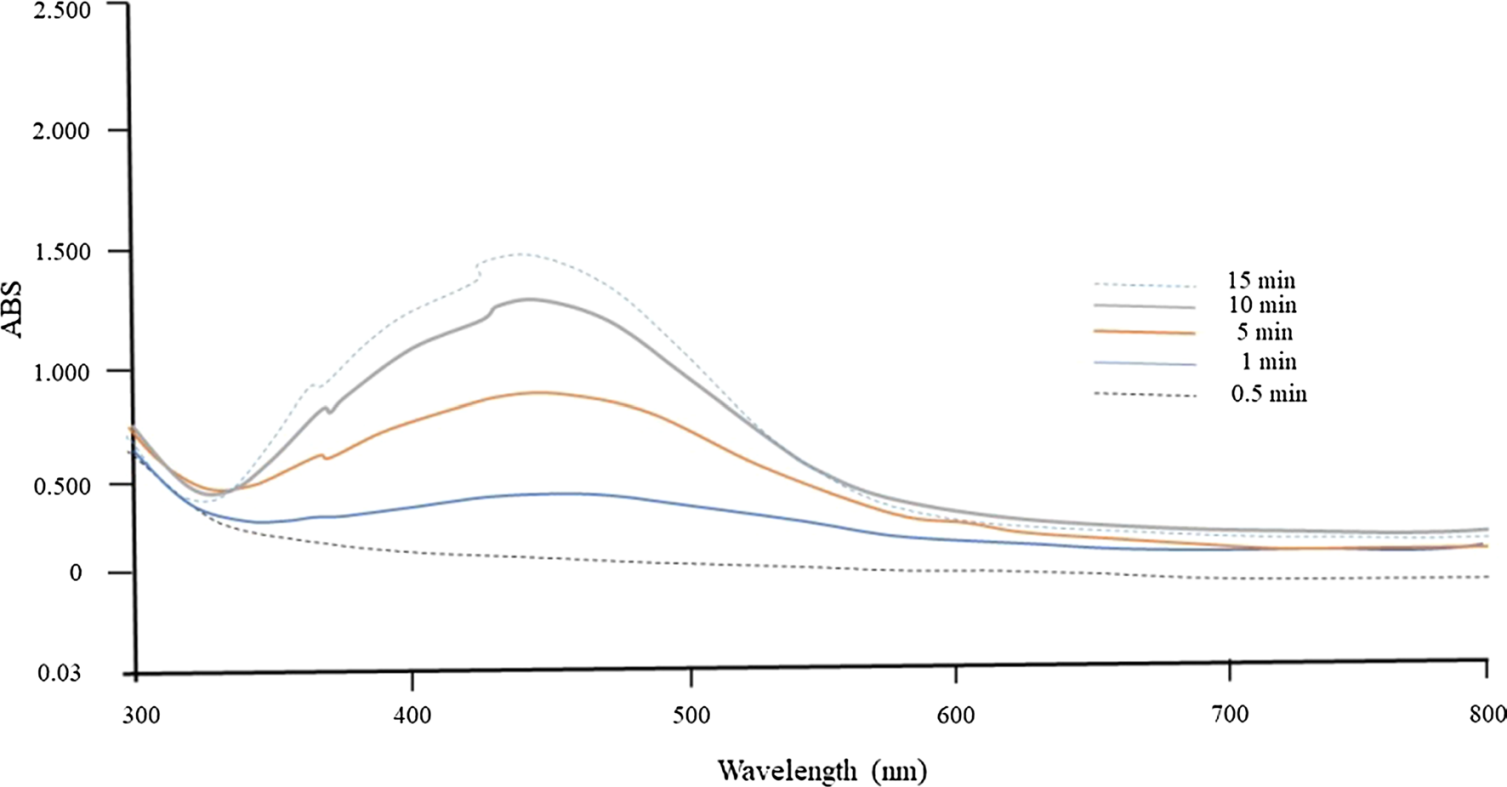
UV–Vis absorption spectra of biosynthesized AgNP*s* from *A. lycoctonum*. The inset shows the changes in absorbance as a function of reaction time

*Aconitum lycoctonum* mediated AgNPs formed by exposure to the equimolar (1 mM) mixture of AgNO_3_ and *A. lycoctonum* polar extract solution (1:1) were monitored for color changes. A strong color change from transparent to light brown and then to a deep reddish brown was observed over the course of 15 min of exposure to diffused sunlight, indicating the formation of AgNP*s* (Fig. 1). This indicates that the minimum time required for complete reduction of silver ions by *A. lycoctonum* is 15 min and no AgNO_3_ is left for further reaction. In addition, the process of AgNP*s* formation was also monitored by UV–Vis spectrophotometry. The AgNP*s* exhibited an SPR absorption peak at 460 nm after 15 min which confirmed the formation of biogenic AgNP*s*. The absorption intensity was also found to increase with time, which was attributed to the continuous reduction of silver ions to AgNP*s* (Fig. 2). These UV–Vis spectroscopic results are in good agreement with the previously reported plant extract mediated and sunlight-assisted synthesis of Ag NP*s* [25, 26].

### Fourier transform infrared spectroscopy

**Fig. 3:**
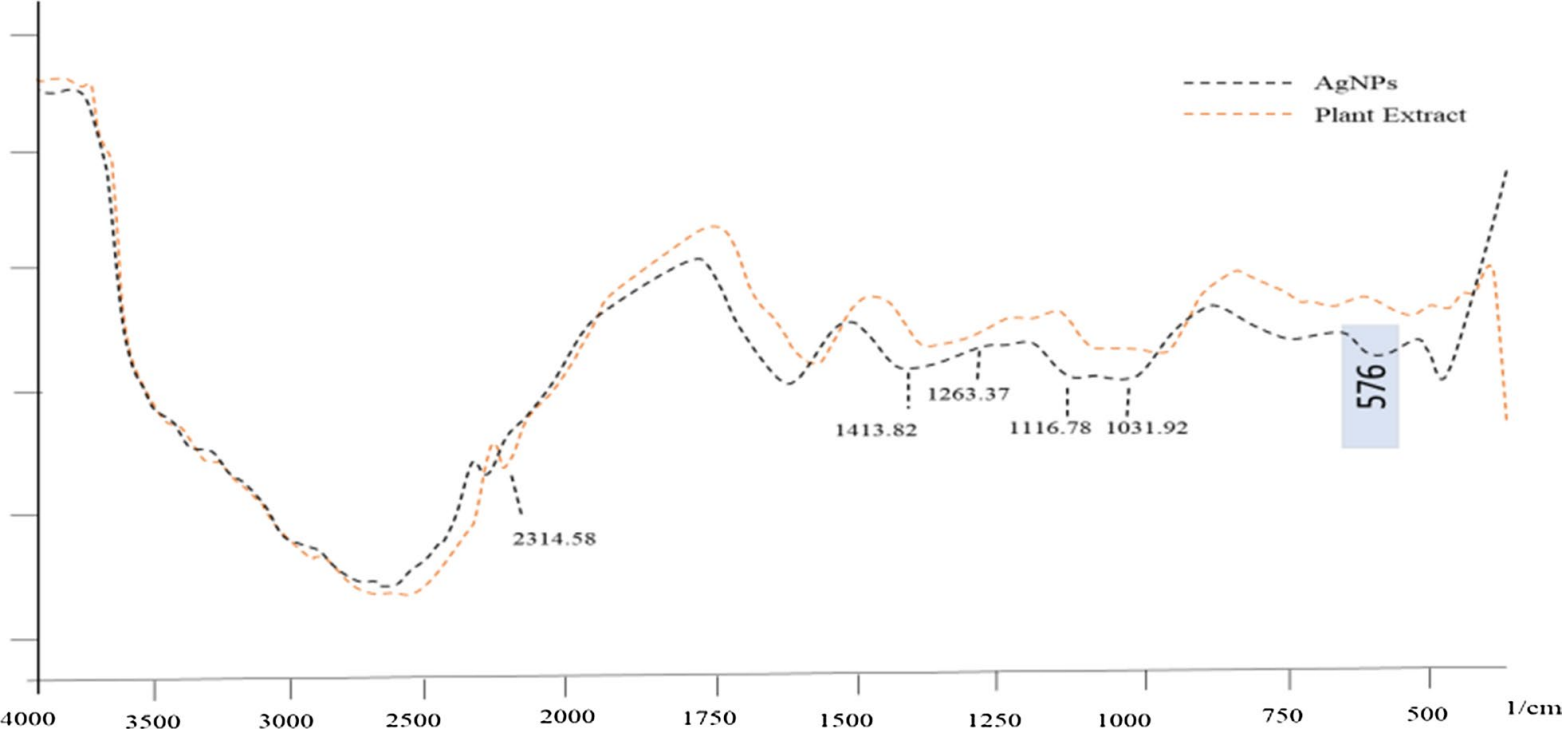
FTIR spectrum of AgNP*s* synthesized under diffuse sunlight after bio-reduction of AgNO_3_ with the polar extracts of *A. lycoctonum*

Identification of the biomolecules and functional groups responsible for stabilization of the freshly prepared AgNPs was performed by FTIR analysis. In the biogenic synthesis of AgNP*s*, the polar extract of the tuberous root of *A. lycoctonum* served as both reducing agent and capping agent. This may be attributed to the presence of some functional groups, which was confirmed by FTIR analysis of both the polar extract of tuber root and the synthesized AgNP*s* (Fig. 3). An absorption band at 576 cm^−1^, which was not present in the polar extract of *A. lycoctonum,* can be associated with the stretching vibrations of Ag–O bonds [27].

However, the overall FTIR spectrum of the AgNP*s* mediated by *A. lycoctonum* and its polar extract is similar, with slight shifts in band positions. The polar extract of tuber roots of *A. lycoctonum* shows bands at 1020.34 cm^−1^, 1076.28 cm^−1^, 1126.43 cm^−1^, 1247.94 cm^−1^, 1625.99 cm^−1^and 2318.44 cm^−1^. Similarly, the FTIR spectrum of *A. lycoctonum* mediated AgNP*s* shows band shifts at 1031.92 cm^−1^, 1116.78 cm^−1^, 1263.37 cm^−1^, 1413.82 cm^−1^, 1629.85 cm^−1^, and 2314.58 cm^−1^. In the spectrum of the polar extract of *A. lycoctonum* and AgNP*s* synthesized with this extract, important changes were detected in the range of 1000 cm^−1^ and 2350 cm^−1^. Our results are in agreement with several recent reports, such as [28, 29].

### Scanning electron microscope

**Fig. 4:**
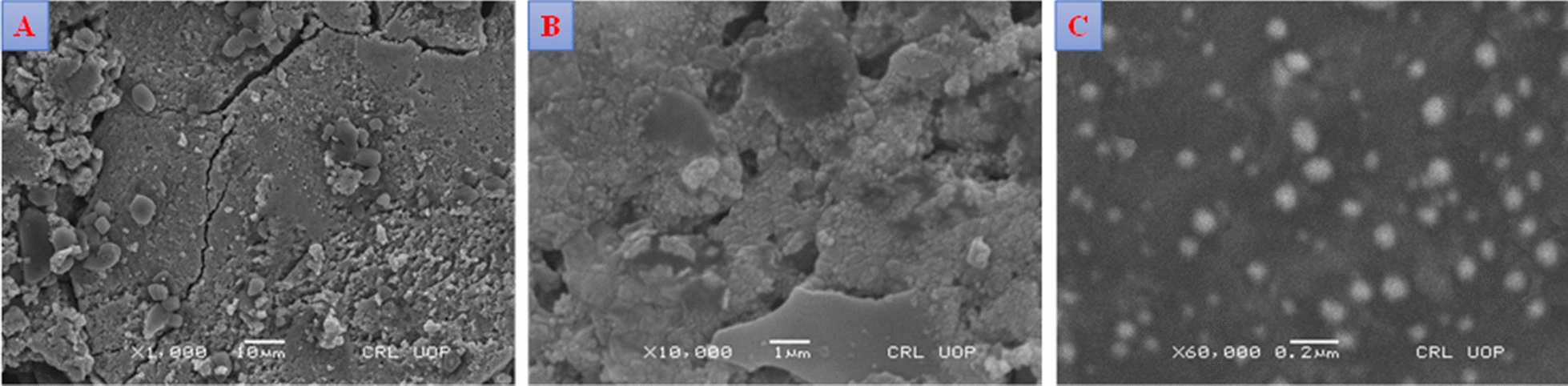
The SEM images of AgNPs synthesized under diffuse sunlight with the polar extract of *A. lycoctonum* polar extract at different magnifications

The surface morphology of the bio-synthesized AgNPs was determined by scanning electron microscopy. Figure 4A–C) shows SEM microscopic images of AgNP*s*. Nanoparticles with nearly identical shapes and dimensions and diameters less than 100 nm with an average size of 67 nm were confirmed by SEM analysis.

### Characterization of AgNP*s* by EDX

**Fig. 5:**
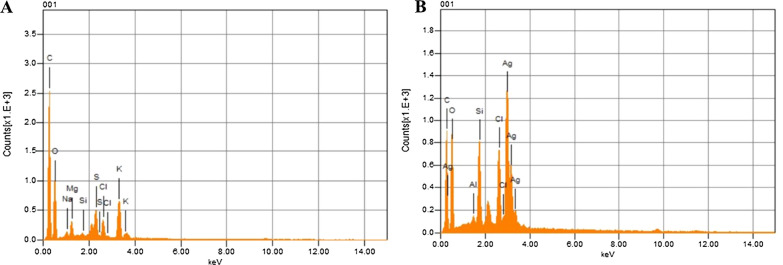
EDX of AgNP*s* synthesized with the polar extract of *A. lycoctonum*

EDX was used to determine the purity of AgNPs by analyzing their elemental composition. Figure 5 shows the graphical representation of the EDX result. Silver is the primary and most prominent peak in the diagram 3.5 (B), which is not present in 3.5 (A). The plot produced also shows the presence of carbon, oxygen, and chlorine, indicating that these elements are present in the plant material. Here, the energy dispersive spectrum indicates that silver is the main component of the synthesized nanoparticles. AgNP*s* generally show a strong typical signal peak at 3 keV, which is due to SPR [30].

### Dynamic light scattering (DLS) measurements

**Fig. 6:**
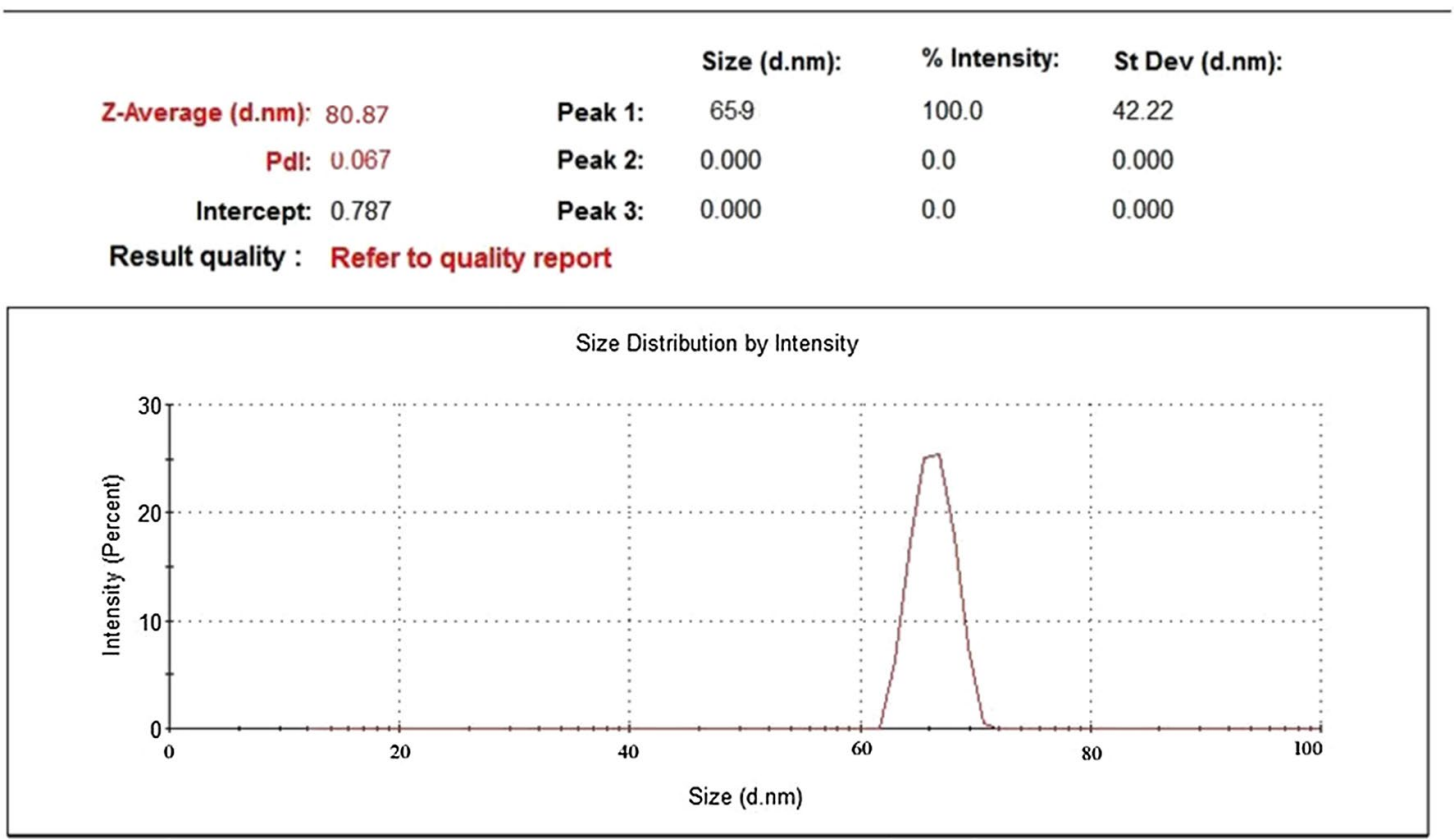
Dynamic light scattering (DLS) image of biogenic AgNP*s*

The z-average for AgNP*s* is shown in Fig. 6. The size of biogenic AgNP*s* was measured by DLS, and the z-average is 65.9 nm. This size of AgNP*s* was in agreement consistent with the size obtained from the measurements of SEM.

### Antibacterial activity

**Fig. 7:**
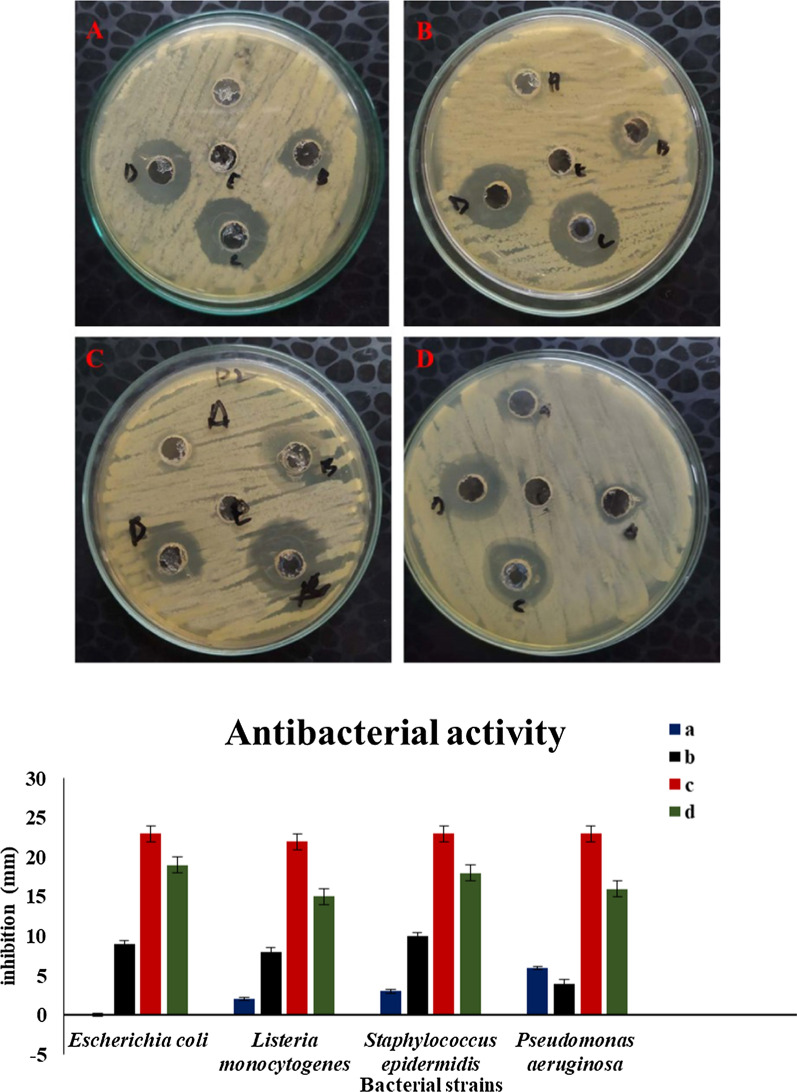
Antimicrobial activity of AgNP*s* against various pathogenic and opportunistic bacterial strains **A** *Escherichia coli,* **B** *Listeria monocytogenes,* **C** *Staphylococcus epidermidis,* **D** *Pseudomonas aeruginosa,* (a) plant extract, (b) AgNO_3_, (c) positive control, (d) AgNP*s*, (e) negative control

Agar well diffusion method was used to investigate the antibacterial activity of AgNP*s* mediated by plant extracts against four different strains of pathogenic and opportunistic bacteria. The evaluation of the antibacterial activity of these AgNP*s* is shown in Fig. 6. The results indicate that these AgNPs are potentially effective in reducing bacterial growth, albeit with varying intensity. The zones of inhibition (ZOI) for AgNPs against *Escherichia coli (ATCC 10536), Listeria monocytogenes (ATCC 13932), Staphylococcus epidermidis (ATCC 12228), and Pseudomonas aeruginosa (ATCC 10145)* were 19, 15, 18, and 16 mm, respectively. *A. lycoctonum* AgNP*s* inhibited the growth of all bacterial strains studied; however, some of the bacteria studied were resistant to the *pure A. lycoctonum* extract. Our results are in close agreement with the standard antibacterial drug ceftriaxone sodium (Fig. 7), demonstrating the efficacy of these nanoparticles against clinically important bacteria.

This property of AgNP*s* to inhibit microbial growth is a consequence of their very small size. The widespread bactericidal/ bacteriostatic activity of AgNP*s* has been well described in previous literature [31]. AgNPs have an antibacterial effect because they bind to the bacterial cell wall. This binding leads to the accumulation of coat protein precursors, resulting in protein denaturation, loss of proton motive force, and eventual cell death [32].

### Antioxidant activity

#### Ferric reducing antioxidant power assay

**Fig. 8:**
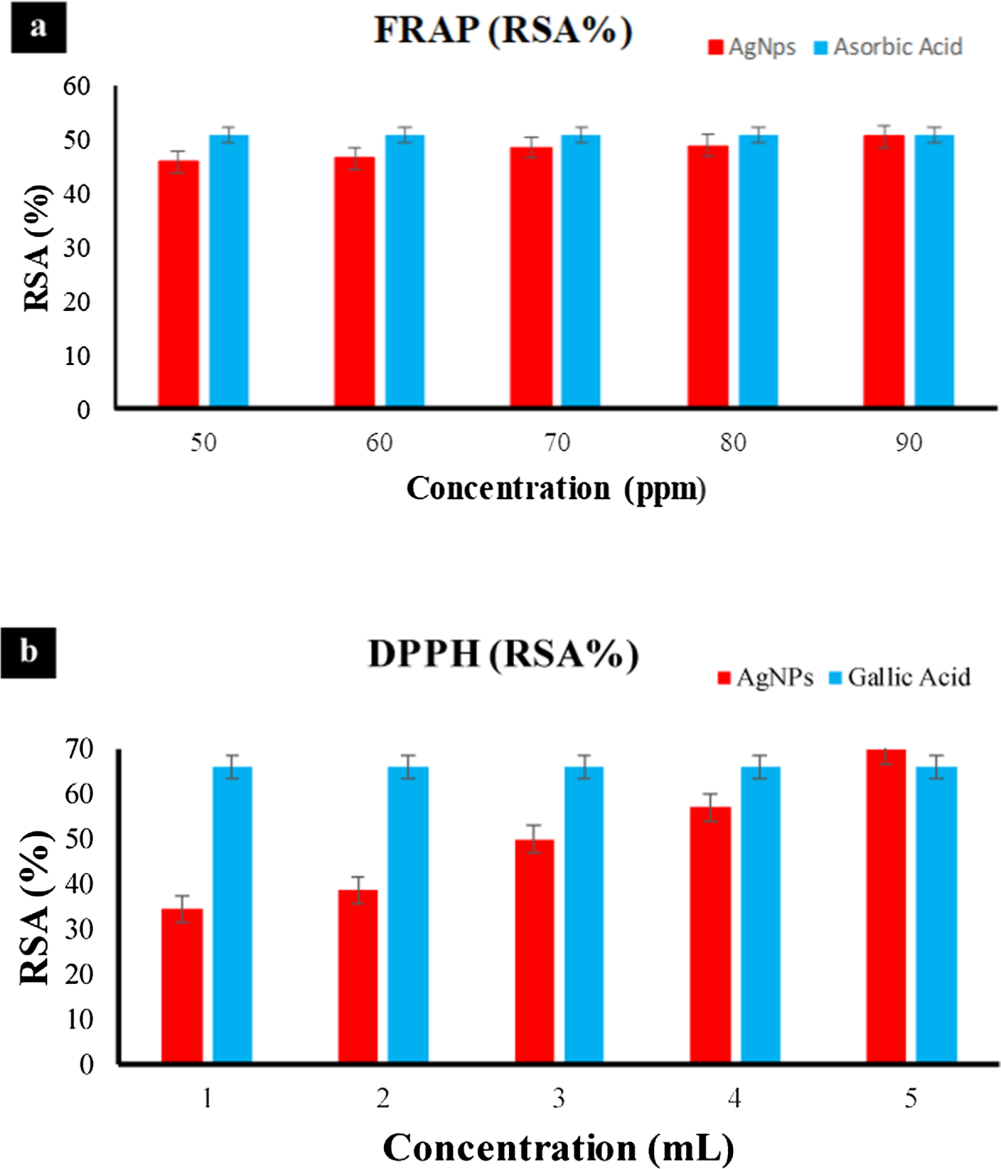
Antioxidant activity of the synthesized AgNP*s*, a-FRAP method b-DPPH method

Total antioxidant activity was measured by the ferric reducing antioxidant assay (FRAP). Since ascorbic acid is a secondary antioxidant that can neutralize free radicals and interrupt chain reactions, it was chosen as the reference solution. The free hydroxyl groups of vitamin C scavenge free radicals, and their antioxidant effect is enhanced by their polyhydroxyl content. Using an ascorbic acid solution as a standard, the antioxidant activity was measured by the FRAP method. To get rid of the deposited potassium ferrocyanide (K_3_Fe(CN)_6_) complex, a solution of trichloroacetic acid (TCA) was used. When FeCl_3_ is added, a complex with a color range from green to blue (blue berlin) is formed. The ability to reduce was a promising marker for an antioxidant substance. In this study, the reducing power was determined by the ability of an antioxidant to reduce Fe^+3^ to Fe^+2^. At a concentration of 50–90 ppm, the percentage of RSA ranged from 45.69 to 50.47% (see Fig. 8). The antioxidant ability of the synthesized nanoparticles can be attributed to stabilization of the radicals by the mechanism of simple electron transfer and -proton transfer, sequential proton loss and electron transfer, or simply by the mechanism of hydrogen atom transfer [33].

#### 2,2-Diphenyl-1-picrylhydrazyl radical scavenging capacity assay

The 50% inhibitory or radical scavenging concentration was used to determine DPPH radical scavenging activity. In the DPPH assay, the AgNP*s* show significant activities but lower compared to the standard. Therefore the, AgNP*s* synthesized from *A. lycoctonum* showed excellent antioxidant potential as shown in the Fig. 8. Our result shows good agreement with a previously reported study [34, 35]. Therefore, *A. lycoctonum*-AgNPs have the potential to be used in both pharmaceutical and food industries.

### Anti-inflammatory activity

#### Inhibition of the protein denaturation

Medicinal plants are believed to be a significant source of novel chemicals with potential therapeutic benefits. The study of plants is therefore considered a fruitful and rational strategy in the search for new anti-inflammatory drugs that are likely to be used as anti-inflammatory agents in folklore. Inflammation can be harmful and lead to hypersensitivity reactions, which can be fatal, and persistent organ damage [36]. NSAIDs are thought to prevent the denaturation of proteins that act as antigens and cause autoimmune diseases. In inflammatory diseases, such as rheumatoid arthritis, protein denaturation is a known cause of the body's inflammatory response [37]. Therefore, the ability of the studied *A. lycoctonum* mediated AgNP*s* to inhibit protein denaturation may explain their anti-inflammatory effects. As can be seen in Table 1*A. lycoctonum* mediated AgNP*s* are responsible for the anti-inflammatory effect in a dose-dependent manner. The value for highest concentration (500 µg/mL) of synthesized AgNP*s* was 91.78% and for the lowest concentration (100 µg/mL) of synthesized AgNP*s* was 51.73%. According to the results of this study, the AgNP**s** were capped by the secondary metabolites of *A. lycoctonum* tuber root extract. The release of lysosomal components by neutrophils at the site of inflammation has been shown to be suppressed by secondary metabolites of plant extracts mediated by AgNP*s*. After their release into the extracellular space, proteinases and bactericidal enzymes stored in lysosomes lead to further inflammation and tissue damage [38]. Similar results were described by [39]. Our results were also in good agreement with previous studies [40].

**Table 1:** Determination of anti-inflammatory activity of AgNP*s* by protein denaturation

Concentration (ppm)	Control (Ac)	Absorbance of sample (As)	% Inhibition
10	0.3126	0.154	50.73
20	0.3126	0.089	71.24
30	0.3126	0.077	75.23
40	0.3126	0.054	82.72
50	0.3126	0.25	91.87

### Anti-diabetic activity

#### α-Amylase inhibition by starch hydrolysis

α-Amylase inhibitors, which prevent starch degradation, are among the popular drugs used to control hyperglycemia and thus find application in the type-2 diabetes mellitus (DM) [41]. By slowing starch digestion, which in turn causes a reduction in glucose absorption in the intestine, α-amylase inhibition is a good strategy for alleviating the symptoms of DM. The primary goal of α-amylase inhibition is to reduce glucose formation, but it can also reduce the action of glucosidase and α-amylase by removing their substrates. Several herbs have been shown to contain antidiabetic properties and therefore used as traditional medicines throughout the world [42].

**Fig. 9:**
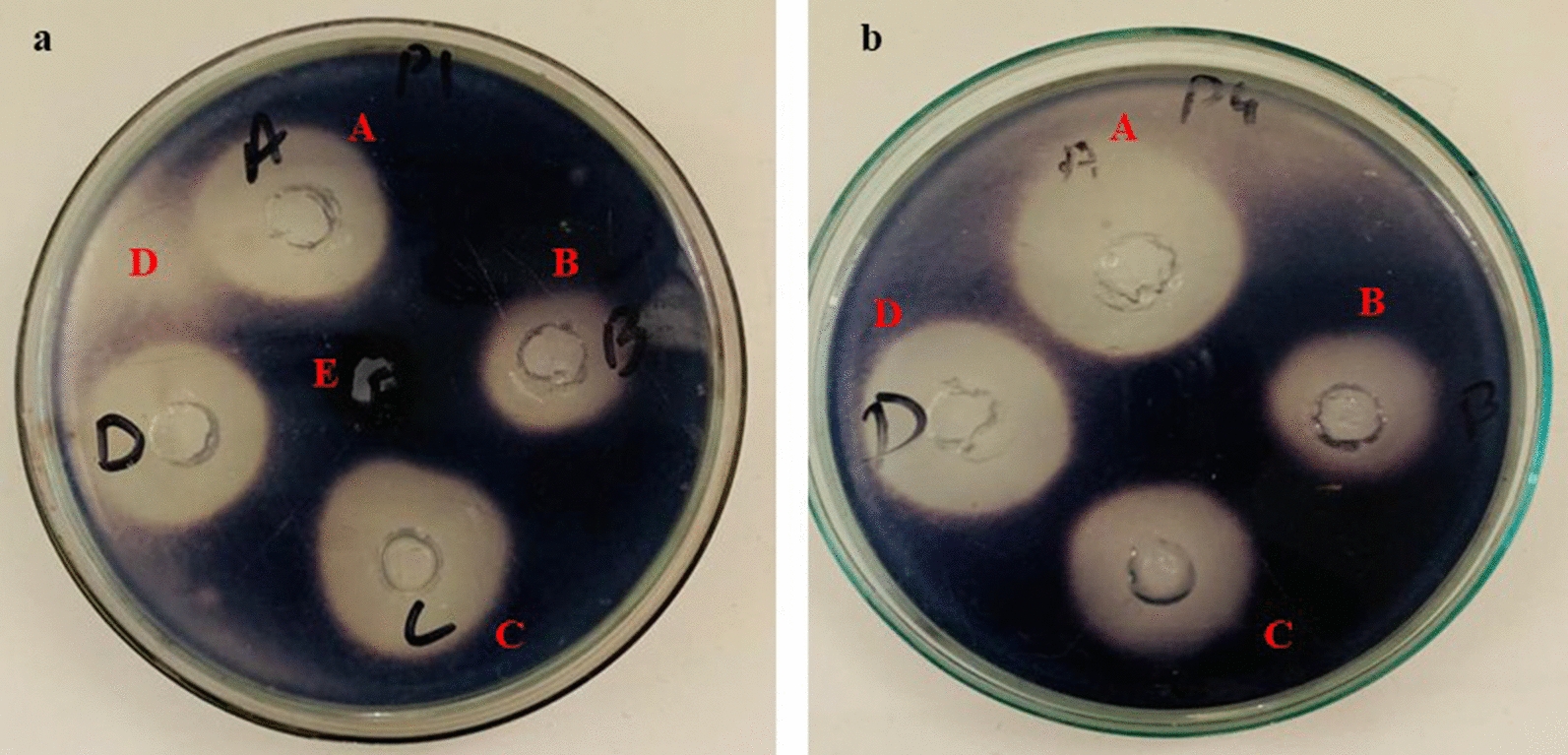
Inhibitory effect of AgNP*s* synthesized from the polar extract of *A. lycoctonum* against α-amylase. **A** Positive control, **B** standard (Acarbose), **C** AgNP*s*, **D** plant extract, **E** negative control

In the present study, the results of preliminary agar diffusion amylase inhibition assays showed that AgNP*s* mediated by *A. lycoctonum* inhibited the α-amylase enzyme, compensating for the hydrolysis of starch, as shown in Fig. 9. The percentage inhibition of each solution is shown in Table 2.

**Table 2:** α-Amylase inhibition by starch hydrolysis

Solution	Concentration (µL)	Zone of inhibition (mm)	% Inhibition
Standard (acarbose)	20	20	33.0
	30	21	34.3
AgNP*s*	20	27	16.6
	30	24	25.0
Control with out	20	32	0
inhibitor	30	32	0

#### Digestive enzymes inhibition and kinetics

DM is a metabolic disease characterized by a constantly high blood glucose level. The initial rise in blood glucose after a meal is due to the body's metabolism of carbohydrates [43]. Inhibitors of the enzymes involved in carbohydrate metabolism, α-amylase and β-glucosidase, are essential for controlling blood glucose levels in people with DM [44]. One such enzyme inhibitor is acarbose, which delays the digestion of carbohydrates, slows glucose absorption, and maintains stable blood glucose levels stable by inhibiting enzyme activity. Inhibition of the major intestinal enzymes, α-amylase and β-glucosidase, was used to evaluate the in vitro antidiabetic effects of plant extracts and synthesized AgNP*s* [45].

**Fig. 10:**
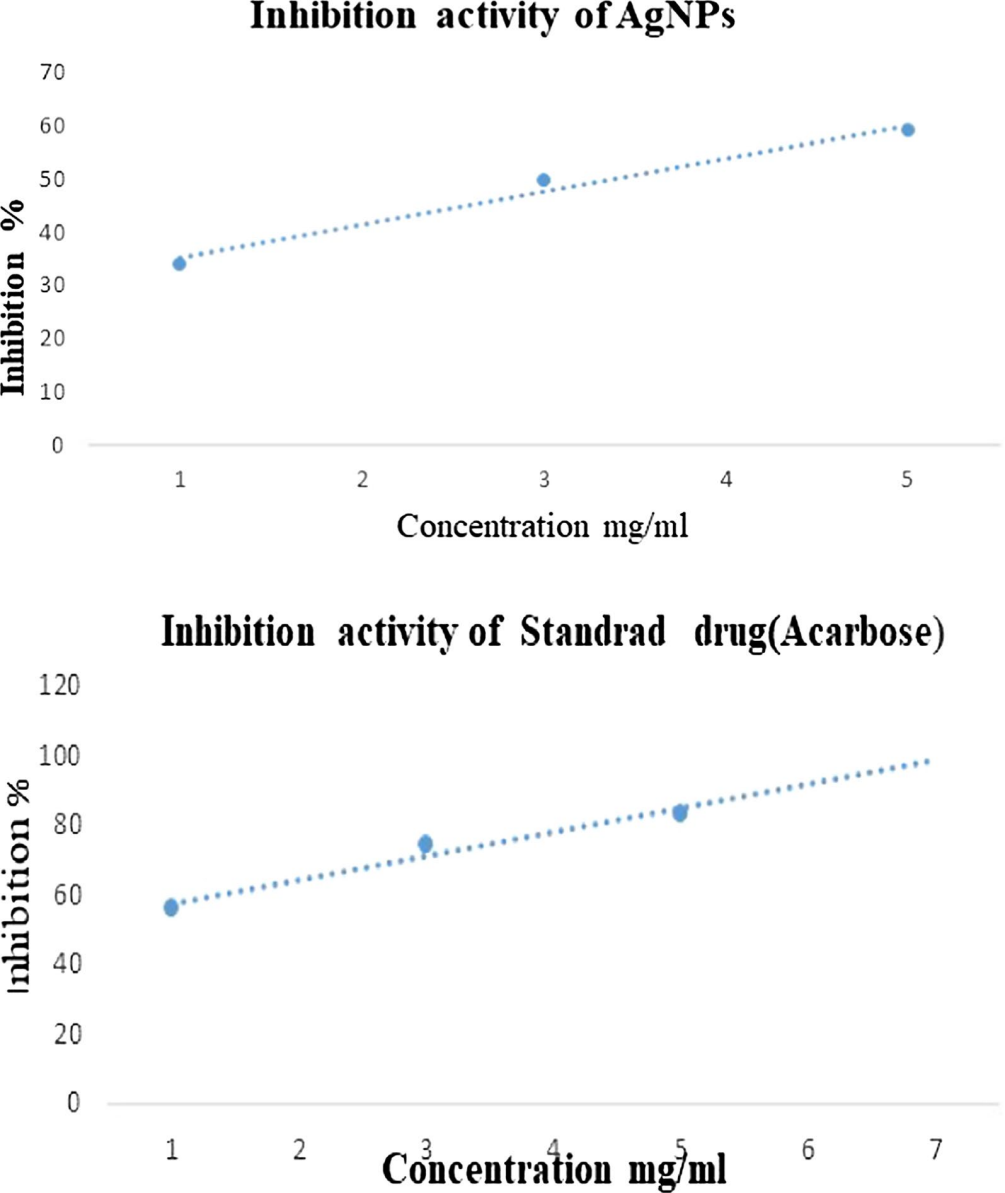
Inhibitory effect of AgNP*s* synthesized from the polar extract of *A. lycoctonum* and the standard drug (acarbose) against α-amylase

AgNP*s* mediated by *A. lycoctonum* showed strong α-amylase activity depending on concentration. Of all the sample solutions, 59.12% inhibitory activity was observed at a concentration 30 mg/mL of the synthesized AgNP*s* (Fig. 10). The lowest inhibitory activity, 34.11% was observed at a concentration 10 mg/mL of the synthesized AgNP*s*. Similarly, the tablets containing acarbose (standard drug) showed the highest inhibitory activity, 83.14% at a concentration of 30 mg/mL. The present study revealed that AgNP*s* mediated by *A. lycoctonum* exhibited potent α-amylase inhibitory activity [46]. The mechanism of mutual interaction between antibiotics and antidiabetic drugs was investigated. Since, *A. lycoctonum-*AgNP*s* have very good antibacterial activity, it is suggested that these AgNPs also have antidiabetic activity. In addition, our results are in agreement consistent with several studies already published in the literature [47, 48].

### Comparison with literature

A comparison of the applications, sizes and sources of the various biogenically synthesized AgNP*s* with the current ones is shown in Table 3.

**Table 3:** Biogenic synthesis of AgNPs using various plant extracts and their applications

Biogenic source	Plant part	Size (nm)	Applications	References
*Ascophyllum nodosum* (L.) Le Jol	Whole plant	69	Antifungal, antioxidant, Cytotoxicity	[49]
*Sida retusa* L	Leaf	75.4	Antimicrobial, catalytic	[50]
*Solanum khasianum* C.B. Clarke	Leaf	26	Antioxidant, antimicrobial, antidiabetic	[51]
*Caesalpinia bonducella* (L.) Fleming	Leaf	50.3	Anti-inflammatory, anti-cancer activities	[52]
*Zephyranthes rosea* Lindl	Flower	30	Anti-inflammatory, antimicrobial, antioxidant activities	[53]

## Conclusion

In this study, a very simple, inexpensive, straightforward, reproducible, and environmentally friendly approach for the synthesis of AgNP*s* under diffused light using polar extract of *A. lycoctonum* as reducing, stabilizing, and capping agent is presented. The formation of AgNP*s* and characterization of the synthesized nanoparticles were carried out by observing the color change, UV–vis spectroscopy, FTIR spectroscopy, SEM and EDX. The AgNP*s* mediated by A. *lycoctonum* have shown very good antibacterial activity by inhibiting the growth of both Gram-positive and Gram-negative bacteria as efficiently as the standard antibacterial drug ceftriaxone sodium. A very good antioxidant potential was shown by these AgNP*s*, as evidenced by the FRAP assay and the DPPH assay. Moreover, the ability of biogenic AgNP*s* to denature ovalbumin showed that these nanoparticles have anti-inflammatory potential. The antidiabetic potential of the *A. lycoctonum*-based AgNP*s* was measured using two different protocols, and it was found that these AgNP*s* inhibited α-amylase very well and therefore could be used for the treatment of diabetes. Hence, the biosynthesized AgNP*s* were found to be multifunctional and showed a high level of antibacterial, antioxidant, anti-inflammatory and antidiabetic potential. Moreover, this green method for synthesizing AgNPs using *A. lycoctonum* is the best method for producing these materials compared to toxic chemical and other physical methods.

## Data Availability

All data generated or analysed during this study are included in this published article.
