# Correction to: *Aconitum lycoctonum* L. (Ranunculaceae) mediated biogenic synthesis of silver nanoparticles as potential antioxidant, anti-infammatory, antimicrobial and antidiabetic agents

**DOI:** 10.1186/s13065-023-01058-2

**Published:** 2023-10-25

**Authors:** Zia ur Rehman Khan, Nasir Assad, Muhammad Naeem-ul-Hassan, Muhammad Sher, Fatema Suliman Alatawi, Mohsen Suliman Alatawi, Awatif M.E. Omran, Rasha M.A. Jame, Muhammad Adnan, Muhammad Nauman Khan, Baber Ali, Sana Wahab, Sarah Abdul Razak, Muhammad Ammar Javed, Alevcan Kaplan, Mehdi Rahimi

**Affiliations:** 1 Institute of Chemistry, University of Sargodha, 40100 Sargodha, Pakistan; 2 Department of Biochemistry, Faculty of Science, University of Tabuk, Tabuk, Saudi Arabia; 3 Department of Pediatrics, College of Medicine, King Saud Bin Abdulaziz University for Health Sciences, Riyadh, Saudi Arabia; 4 Department of Chemistry, Faculty of Science, University of Tabuk, Tabuk, Saudi Arabia; 5 Department of Chemistry, Faculty of Education, University of Dalanj, Dalanj, Sudan; 6 Department of Chemistry, Islamia College Peshawar, 25120 Peshawar, Pakistan; 7 Department of Botany, Islamia College Peshawar, 25120 Peshawar, Pakistan; 8 Department of Plant Sciences, Quaid-I-Azam University, Islamabad, Pakistan; 9 Institute of Biological Sciences, Faculty of Science, Universiti Malaya, Kuala Lumpur, 50603 Malaysia; 10 Institute of Industrial Biotechnology, Government College University, 54000 Lahore, Pakistan; 11 Department of Crop and Animal Production, Sason Vocational School, Batman University, 72060 Batman, Turkey; 12 Department of Biotechnology, Institute of Science and High Technology and Environmental Sciences, Graduate University of Advanced Technology, Kerman, Iran

**Correction to: BMC Chemistry (2023) 17:128**

10.1186/s13065-023-01047-5

Following publication of the original article [1], the author name “Fatema Suliman Alatawi” was incorrectly written as “Suliman Alatawi”. This has been corrected with this erratum.

The original article has also been corrected.
